# Impact of Surface States in Graphene/*p*-Si Schottky Diodes

**DOI:** 10.3390/ma17091997

**Published:** 2024-04-25

**Authors:** Piera Maccagnani, Marco Pieruccini

**Affiliations:** 1 Consiglio Nazionale delle Ricerche, Istituto per la Microelettronica e i Microsistemi, Via P. Gobetti 101, 40129 Bologna, Italy; 2 Department of Physics and Earth Sciences, University of Ferrara, Via Giuseppe Saragat 1/c, 44122 Ferrara, Italy

**Keywords:** graphene/silicon Schottky diode, surface state, diode parameter extraction, modeling, charge transport, 2D materials, Fermi liquids

## Abstract

Graphene–silicon Schottky diodes are intriguing devices that straddle the border between classical models and two-dimensional ones. Many papers have been published in recent years studying their operation based on the classical model developed for metal–silicon Schottky diodes. However, the results obtained for diode parameters vary widely in some cases showing very large deviations with respect to the expected range. This indicates that our understanding of their operation remains incomplete. When modeling these devices, certain aspects strictly connected with the quantum mechanical features of both graphene and the interface with silicon play a crucial role and must be considered. In particular, the dependence of the graphene Fermi level on carrier density, the relation of the latter with the density of surface states in silicon and the coupling between in-plane and out-of-plane dynamics in graphene are key aspects for the interpretation of their behavior. Within the thermionic regime, we estimate the zero-bias Schottky barrier height and the density of silicon surface states in graphene/type-*p* silicon diodes by adapting a kown model and extracting ideality index values close to unity. The ohmic regime, beyond the flat band potential, is modeled with an empirical law, and the current density appears to be roughly proportional to the electric field at the silicon interface; moreover, the graphene-to-silicon electron tunneling efficiency drops significantly in the transition from the thermionic to ohmic regime. We attribute these facts to (donor) silicon surface states, which tend to be empty in the ohmic regime.

## 1. Introduction

For more than five decades, silicon (Si) has been the principal material for microelectronics thanks to its simple production, low cost and well-defined processing routes. But in the last 15 years, two-dimensional (2D) materials (TDMs) have taken a lot of attention as possible candidates for next generation electronics, promising completely novel devices and a huge impact on several markets [1]. Among others, graphene (Gr) is particularly attractive because of its excellent electronic properties and because it can be easily integrated into the standard CMOS (Complementary Metal-Oxide Semiconductor) process, using a back-end-of-line approach. Considering the devices, the Gr/Si Schottky junction has received a lot of attention, being largely used in different fields of applications, such as biological and chemical sensing, or as photodetectors, and because it is one of the simplest conceivable interfaces for the development of the future generation of electronic and optoelectronic devices [2]. Therefore, it is important to achieve a deep understanding of the main physical mechanisms that govern the current transport in these devices, constructing a model which would aid in predicting their electrical behavior.

The Gr/Si structures show rectifying properties similar to ordinary 3D/3D metal/semiconductor Schottky diodes, and the methods originally developed for the latter are often used to probe the features of the former through, e.g., the analysis of the current/voltage (*I*-*V*) behavior at different temperatures (known as Richardson analysis). But with graphene, some main important differences cannot be overlooked, such as (i) the dependence of the Gr Fermi level on the carrier density [2,3] and (ii) the peculiarities in the charge transfer mechanism between a 2D and a 3D material in a vertical configuration, responsible for the rather low values of the Richardson constant found for these devices [4].

The last point has been thoroughly considered by Ang et al. [5,6] for Gr/*n*-type semiconductor devices, and recent experimental observations have been discussed also in the light of those results [4]. A further issue that seems not to have drawn enough consideration in the literature is the role played by the semiconductor surface states. Their density may be not negligible at all with respect to the amount of carriers in Gr, or, among the latter, of just those which succeed in tunneling across the interface. Again, the rather low values observed for the Richardson constant strongly motivate us to pay attention to this topic, suggesting that the way the localized states affect the performances of these systems may differ from what is expected in 3D/3D devices. The role of silicon surface states has been extensively investigated in the past, particularly within the MOS community, as current conduction and other device characteristics in MOS transistors were largely influenced by surface or interface states. However, when considering a Gr/Si structure, we encounter a fundamentally different situation compared to MOS transistors. In this case, the 2D graphene layer is mechanically transferred onto the silicon substrate and does not form a strong physical bond with it; rather, it is attached by van der Waals forces. A potential model for the interaction between graphene and silicon is proposed in [7], along with a description of how surface defects could impact current conduction in Gr/Si junctions, both under forward and reverse biases. Following this approach, we decided to account for the surface states in the direct fitting of the *I*-*V* characteristics of Gr/type-*p* silicon (Gr/*p*-Si) diodes, both in the thermionic and in the ohmic regime, i.e., beyond the flat band potential, in order to further provide some evidence of their effect. The current–voltage characteristic of a Schottky junction derived in the regime of thermionic emission for 3D/3D metal/type-*p* semiconductor Schottky devices [2,8] is

(1) $$I=I_{0}\;e^{-qV_{bias}/\left(nk_{B}T\right)}\left[1-e^{qV_{bias}/k_{B}T}\right],$$

expressing the current *I* as a function of the bias potential $V_{bias}$ applied to the metal relative to the semiconductor (note that, as a consequence, the diode is directly polarized for $V_{bias}<0$, with *q* being the absolute value of the electron charge). In Equation (1), the ideality factor *n* (≳1) implicitly accounts for surface states, image charge effects, band curvature and interfacial inhomogeneities;

(2) $$I_{0}\equiv A\;A^{*}T^{2}e^{-\Phi_{B0}/k_{B}T}$$

is the saturation current, where *A* is the diode active area, $A^{*}$ is the Richardson constant [2] and $\Phi_{B0}$ is the zero-bias Schottky Barrier Height (SBH).

In this paper, we study the kinetics of charge carriers across the graphene–Si interface in vertical Schottky diodes introducing the probability for a particle to abandon Gr and the transmission coefficient T for the evaluation of incident Gr electrons. Following this approach, the factor $A^{*}T^{2}$ in Equation (2) is here replaced with an expression derived in the theory section by qualitative arguments based on the Landau theory of the Fermi liquid [9]. This kind of an approach is inspired by the work of Trushin [10] on Gr/type-*n* semiconductor Schottky diodes. Moreover, we explicitly introduce the effect of the silicon surface states through the built in potential $\phi_{bi}\left(V_{bias}\right)$, in the Arrhenian dependence $\exp\{-\left[\Phi_{B0}+qV_{bias}/n\right]/k_{B}T\}$ of Equations (1) and (2) following the model proposed by Zhong [11]. In the ohmic regime, an empirical fitting expression will be provided; this suffices to highlight the changes observable in the tunneling efficiency with respect to the thermionic case.

## 2. Theory

We consider an ideal system consisting of a *p*-doped Gr sheet separated from a plane *p*-Si surface by a thin native oxide layer, in the order of 1 nm thick. Aimed at dealing with a model, homogeneous interfaces are assumed. The oxide layer sustains a potential difference that affects the dependence of the built-in potential on the applied bias. This point is explicitly considered below in the balance equation relating the bias potential with the Fermi levels of Gr and *p*-Si. As a simplifying assumption, donor surface states located just at the Si/oxide interface are envisaged, yet their wave functions penetrating within the oxide layer.

The electron population in Gr is conveniently described as an ensemble of quasi-particles (or their anti-particles [9,12]), which represent delocalized elementary excitations of a Fermi liquid (a bosonic analog can be viewed in the more familiar case of phonons in a lattice). It is possible to speak of such states if their energy and momentum are large with respect to the corresponding quantum uncertainties [9]; this condition leads to the inequality $\left|E_{Fg}\right|\gg k_{B}T$, i.e., the electron distribution must be weakly smoothed about the Fermi level (e.g., referred to the Dirac point).

### 2.1. Quantum Estimates on Charge Transport

In a free standing Gr single layer, electrons behave as massless particles with respect to the in-plane dynamics. This is not the case with regards to the direction perpendicular to the sheet [5], where the electron is confined in a ∼0.3 nm thick layer. The kinetic energy contribution inherent to this localization can be roughly estimated via the Heisenberg relation $\Delta p_{⊥}\Delta r_{⊥}∼\hbar$ as [13]

(3) $$E_{kin,⊥}∼\frac{\hbar^{2}}{2m\Delta r_{⊥}^{2}}\;,$$

where $\Delta p_{⊥}$ and $\Delta r_{⊥}$ are the momentum and position uncertainties perpendicular to the Gr sheet, *ℏ* is the reduced Planck’s constant, and *m* is the electron mass. With $\Delta r_{⊥}∼0.3$ nm, one has $E_{kin,⊥}∼0.5$ eV.

The $r_{⊥}$ localization in Gr follows from Coulomb interaction with the nuclei, but it can be pictured as a confinement in a potential well that could be assumed to be $U_{0}∼10\;E_{kin,⊥}$ deep. Thus, one gets a value close to the Gr work function, which is indeed the barrier height adopted in ref. [5] for a similar description.

In such a system, collisions of electrons with other electrons or phonons/impurities/defects are responsible for a coupling between in-plane and out-of-plane dynamics, representing at the same time the origin of the uncertainty in the energy *E* of the quasi-particles. The latter populate an energy interval $\left|E_{Fg}-E\right|≲k_{B}T$ and their lifetime τ, due to electron/electron interaction, can be derived by simple considerations based on energy scales [12], leading to $\tau ∼\hbar E_{Fg}/{\left(k_{B}T\right)}^{2}$ and to an energy uncertainty in the order of $\Delta E_{\tau }∼\hbar /\tau ∼{\left(k_{B}T\right)}^{2}/E_{Fg}$. This estimate is appropriate when the Gr Fermi level is far enough from the Dirac point [10,14], which is indeed our case (see below).

The possibility that an electron leaves Gr is linked to a non-zero $\Delta E_{\tau }$. Let ${\hat{\epsilon }}_{coll}$ be the perturbation to the Hamiltonian related to collisions, and let $\epsilon_{fi}=\langle f|{\hat{\epsilon }}_{coll}|i\rangle$ be a typical matrix element between an initial state |i〉 and a final one |f〉 where one of the quasi-particles has boosted towards an out-of-plane drift; then, $\Delta E_{\tau }∼\epsilon_{fi}$ and the chance for a quasi-particle to abandon Gr may be estimated as roughly

(4) $$\lambda ≲\frac{\Delta E_{\tau }}{v_{F}\Delta p_{coll}}∼\frac{k_{B}T}{E_{Fg}}\;,$$

where $v_{F}≃10^{8}$ cm/s is the Fermi velocity [2] and $v_{F}\Delta p_{coll}∼k_{B}T$, the typical energy exchange in a collision [9], assures that the quasi-particle remains in the smoothed domain of the Fermi distribution. This condition is essential: for a T=0 distribution, the collisional interaction would be inhibited by the Pauli principle. Equation (4) provides just an order of magnitude for λ; the actual value would increase adding heterogeneous collisions. Note that the condition by which quasi-particles have physical meaning implies λ≪1.

**Figure 1:**
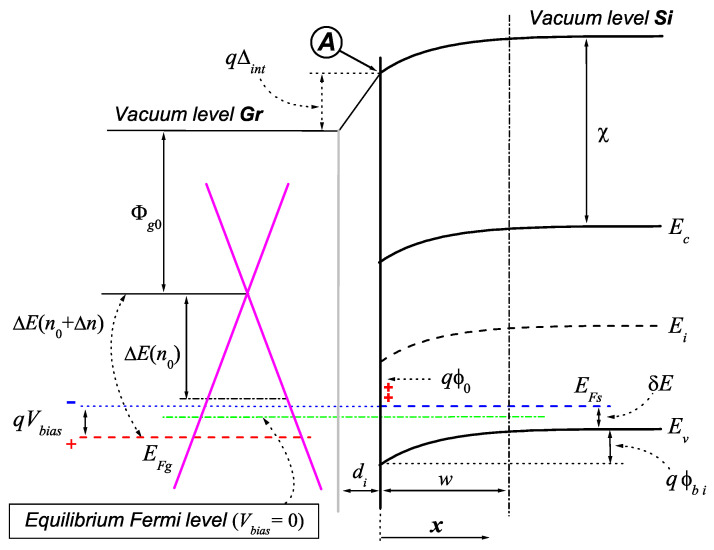
Energy diagram of the Gr/oxide/*p*-Si system under moderate reverse bias ($V_{bias}>0$). $E_{c}$, $E_{i}$, $n_{0}$ and *w* are the bottom energy of the conduction band, the intrinsic Fermi level, the carrier density in Gr at $V_{bias}=0$ and the width of the space charge region, respectively; all other symbols are defined in the text. When $V_{bias}\neq 0$ both $E_{Fg}$ and $E_{Fs}$ shift with respect to the common value at $V_{bias}=0$. Donor surface states are characterized by a constant energy density, and $q\phi_{0}$ is the maximum value of their energy spectrum. A $d_{i}$-thick oxide layer is also represented. Point **A** is a useful reference for the derivation of Equation (8).

In a vertical, stacked configuration, typical for Gr/Si diodes, the presence of an oxide layer between Gr and Si is expected to lower $U_{0}$ significantly at the interface (cf. Figure 1); the presence of localized surface states also relates to this lowering. As often done in the literature, we may model this lowered barrier with a square profile of effective height *U* over the whole thickness $d_{i}$ of the oxide layer. Then, when $V_{bias}=0$, the transmission coefficient T for incident Gr electrons is in the order of [13] (and refs. [15,16] for the relevance of collisions)

(5) $$T\approx e^{-4d_{i}{\left[2m(U-\epsilon )\right]}^{1/2}/\hbar }\;,$$

where ϵ is the total electron energy. Equation (5) has meaning provided the arrival state at the Si side is already empty.

The fraction κ of Gr electrons close to $E_{Fg}$ which may recombine with a hole at the *p*-Si surface reads as

(6) $$\kappa \approx \lambda \;T\;e^{-\Phi_{B0}/k_{B}T}\;,$$

where $e^{-\Phi_{B0}/k_{B}T}$ is the probability to have a hole at the Si surface ($V_{bias}\equiv 0$).

Following this approach, the pre-exponential factor of the saturation current in Equation (2) can be replaced as

(7) $$A^{*}T^{2}⟷\;q\;\frac{n_{0}}{l_{⊥}}\;\lambda \;T\;{\bar{v}}_{⊥}\;,$$

where $n_{0}$ is the carrier surface density in Gr at zero-bias, $l_{⊥}$ is the thickness of the Gr moiety, and ${\bar{v}}_{⊥}$ is the typical out-of-plane carrier velocity. We point out that a tunneled electron emitted from Gr may have undergone interactions which render its $v_{⊥}$ at the Si-side distributed with a certain probability profile.

A number of simplifying, tacit assumptions inhere in the replacement of $A^{*}T^{2}$ in Equations (1) and (2) after Equation (7) (cf., e.g., ref. [17] for a similar discussion). The expression of κ has been derived for $V_{bias}=0$; thus, a correction to *U* due to a non-zero electric field when $V_{bias}\neq 0$ would in principle be more appropriate. Also, the direct tunneling across the Schottky barrier and the electron reflection on the *U*-edge have not been accounted for. However, a refinement of the model which includes these corrections would not improve our analysis, mainly due to the quantum estimates being at most semi-quantitative.

The issue of the surface states needs some consideration. Allowing for localized states also *within* the oxide layer or at the Gr side may possibly influence the effective potential barrier *U*, but apart from a rough estimate of this parameter in the discussion of our data (estimate that is done by assuming an oxide thickness of 1 nm anyway), this is of no relevance to our main focus. On the other hand, we point out that the fraction κ of Equation (6) has been derived in the hypothesis that the surface states are mostly filled; for this reason, they do not contribute to the expression of T. On the other hand, these states are mostly empty in forward polarization when the ohmic regime is entered and should be accounted for. Nevertheless, we shall limit ourselves to discuss our data in terms of the product $\lambda \;T\;v_{⊥}$ irrespective of its detailed expression and show how this quantity changes when the thermionic regime is abandoned to enter the ohmic one. These calculations are deferred to future work.

As a final comment, we note that, being λ∝T after Equation (4), the explicit temperature dependence of κ also conforms to the $T^{1}$ scaling of refs. [4,6].

### 2.2. Thermionic Regime

Electron/hole pairs forming at the Si surface are effective to charge transport if a sufficient energy barrier is crossed. Its height depends on the applied bias ($V_{bias}$) and is influenced by the Si surface states. Assuming temporarily that the transmission coefficient T≡0 (i.e., that the system is a perfect capacitor at equilibrium), the dependence of the electron/hole barrier on the polarization follows from the relation $E_{Fg}=E_{Fs}-qV_{bias}$, where $E_{Fg}$ and $E_{Fs}$ are the Fermi energies in Gr and *p*-Si, respectively. Adopting a common reference (e.g., point **A** in Figure 1), one immediately obtains the balance equation

(8) $$\Phi_{g0}+\Delta E+q\Delta_{int}=\chi +E_{gap}-\delta E-q\phi_{bi}+qV_{bias}\;,$$

where

(9) $$\delta E=k_{B}T\ln\left(N_{v}/N_{a}\right)$$

is the difference between $E_{Fs}$ and the top of the Si valence band edge $E_{v}$, being $N_{v}=1.04\times 10^{19}$ ${\mathrm{cm}}^{-3}$ the effective density of states in the valence band of Si [18] and $N_{a}$ the donor density (≈$10^{15}$ ${\mathrm{cm}}^{-3}$ for the devices considered in this paper); χ=4.05 eV and $E_{gap}=1.12$ eV are the affinity and energy gap of Si, respectively; finally, $\Phi_{g0}≃4.5$ eV is the work function of graphene with respect to the Dirac point [4]. On the other hand, the built-in potential $\phi_{bi}$, the energy ΔE separating $E_{Fg}$ from the Dirac point and the potential $\Delta_{int}$ across the oxide layer all depend on the number Δn of electrons that are exchanged between Gr and Si (by convention Δn>0 if they leave Gr) to establish equilibrium at a given $V_{bias}$. Surface states, which may host or release electrons, play an important role in this redistribution.

Following refs. [19,20], it is assumed a distribution of localized donor surface states with uniform density $D_{i}$ relative to *both* surface area and energy. Let $\Delta E_{0}\equiv {\left[q\phi_{0}-E_{Fs}\right]}_{FB}$ be the maximum energy limit to the surface states with respect to $E_{Fs}$ in the virtual non-equilibrium condition of flat bands; then, surface state electrons with energy $>E_{Fs}$ add up to Δn ones forming a depletion layer with the built-in potential given implicitly by [11]

(10) $$Q_{g}+Q_{s}=\mathrm{sign}\left(\phi_{bi}-\frac{k_{B}T}{q}\right){\left[2q\varepsilon_{s}N_{a}\left|\phi_{bi}-\frac{k_{B}T}{q}\right|\right]}^{1/2}\;,$$

where $Q_{g}\equiv q\Delta n$, $Q_{s}=qD_{i}\left(q\phi_{0}-E_{Fs}\right)$ is the overall positive charge at the empty surface states (cf. the red crosses in Figure 1), and $\varepsilon_{s}$ is the Si dielectric permittivity.

For the remaining terms,

(11) $$\Delta E=\hbar v_{F}\pi^{1/2}\sqrt{n_{0}+\Delta n}\;,$$

with $n_{0}$ the initial doping of graphene [2] and, by Gauss theorem, $\Delta_{int}$ can be expressed as

(12) $$\Delta_{int}=\frac{Q_{g}}{\varepsilon_{d}}\;d_{i}\;,$$

where $\varepsilon_{d}$ is the dielectric permittivity of the oxide layer (which is assumed to be the same as for vacuum).

Equations (8)–(12) provide Δn as a function of $V_{bias}$; when T=0 is relaxed, this relation will be assumed still approximately valid due to the low current densities considered.

The SBH is the energy barrier that a valence electron at $E_{v0}\equiv {\left.E_{v}\right|}_{x=0}$ has to surmount to reach $E_{Fg}$ in order to drift towards Gr due to the electric field, and can be expressed as (cf. Figure 1)

(13) $$\Phi_{B}=\delta E+q\phi_{bi}\left(V_{bias}\right)-qV_{bias}\;.$$

Because this barrier refers to a negative current, we call $\Phi_{B}$ the *backward* SBH.

On the other hand, the term $\exp\{-\left[\Phi_{B0}-qV_{bias}/n\right]/k_{B}T\}$ multiplying the square brackets in Equation (1) is the first order Taylor expansion of

(14) $$\Psi_{B}\equiv \Phi_{B}+qV_{bias}\;=q\left(E_{Fs}-E_{v0}\right),$$

and we refer to $\Psi_{B}$ as to the *forward* SBH.

Now, all the ingredients for the analysis of the *I*-*V* behavior for Gr/Si Schottky diodes have been introduced. Anyway, it is important to underline that the above equations maintain their validity only within a limited $V_{bias}$ interval. Indeed, by increasing the forward polarization, the thickness of the depletion layer progressively decreases until the flat band potential $V_{FB}$ is reached. Then, upon further increasing $\left|V_{bias}\right|$ the ohmic regime is entered, the built-in potential becomes negative, the holes tend to accumulate at the *p*-Si surface [20], and the conduction mechanism in the junction is dominated by the diode series resistance. On the other hand, at sufficiently large reverse $V_{bias}$, the intrinsic Fermi level $E_{i}$ approaches $E_{Fs}$ closely, and an inversion layer forms at the *p*-Si surface, so that the conduction mechanism, still thermionic in nature, differs from that previously described.

While we shall treat the latter in a future work, a brief illustration of the ohmic regime is presented below.

### 2.3. Ohmic Regime

For $|V_{bias}\left|>\right|V_{FB}|$, holes that accumulate at the Si surface [20] are available for recombination with tunneled electrons. Let $\Delta n_{h}$ be the overall excess (surface) hole density with respect to the bulk value; its dependence on $V_{bias}$ can be derived starting from the solution of the one-dimensional Poisson equation for the potential ϕ with an excess positive charge,

(15) $$\rho \left(x\right)=qN_{a}\left\{\exp\left[-\frac{q\phi (x)}{k_{B}T}\right]-1\right\}$$

as a source term (minority carriers are neglected in view of our rough empirical modeling). With the conditions ϕ→0 and E=−dϕ/dx→0 for x→∞, one finds [20]

(16) $$-E=\frac{d\phi }{dx}=\sqrt{\frac{2N_{a}k_{B}T}{\varepsilon_{s}}\left[\frac{q\phi }{k_{B}T}+\exp\left(-\frac{q\phi }{k_{B}T}\right)-1\right]}\;.$$

The excess hole density follows from Gauss theorem

(17) $$\frac{q\Delta n_{h}}{\varepsilon_{s}}={\left.\frac{d\phi }{dx}\right|}_{x=0^{+}}$$

(the half-space x≤0 encompasses the charges in Gr, in the oxide layer and at the Si surface). The dependence $\Delta n_{h}\left(V_{bias}\right)$ follows from Equation (8) and the last two. Then, in order to explore some feature of the conduction mechanism, we adopt for the current the empirical power law

(18) $$I=AJ{\left(\frac{\Delta n_{h}}{n_{0}}\right)}^{\alpha }\;,$$

where *A* is the active area of the diode, and the adjustable parameters are the current density *J* and the exponent α. This dependence fits well the experimental data in the interval $\left[-0.6\;V,\;V_{FB}\right]$, within which $|\Delta n_{h}|≲n_{0}/10$ always holds (see below); moreover, values α≠1 but close to unity will be found.

Since $n_{0}\lambda \;T\ll \left|\Delta n_{h}\right|$, as we shall see, the current in the diode is not limited by the number of available holes at the *p*-Si surface. This supports our assumption regarding the reliability of the balance condition in Equation (8), also in forward polarization.

## 3. Materials and Methods

### 3.1. Device Fabrication

Gr/Si Schottky junctions are fabricated starting from a low-doped *p*-Si substrate with a resistivity of 10–20Ω cm. A 120nm thick silicon oxide was deposited on the wafer by CVD as an insulating layer. The device active areas are lithographically defined, and the ${\mathrm{SiO}}_{2}$ oxide is etched in a standard BOE solution. The trench areas are further cleaned by hydrofluoric acid immediately before the Gr transfer process to limit the formation of the native oxide. A 3×3.5 ${\mathrm{cm}}^{2}$ Gr sheet was transferred onto the Si substrate by a semi-dry method (details are reported in [21]), obtaining a matrix of 100 circular diodes with a radius spanning from 200 to 500 μm. The devices are identified by a capital letter, indicating the row of the matrix, and a number indicating the column.

### 3.2. Characterization

The *I*-*V* measurements are performed at wafer level in a Karl Süss probe station, in ambient atmosphere and in the dark, using four Keithley 238 (KeithIey Instruments, Inc., Cleveland, OH, USA) source monitor units (SMU) connected to the probe through the switching matrix Keithley 707 (with 7072 and 7174 semiconductor cards). As convention, the bias voltage is applied to graphene, while the silicon substrate is grounded in all measurements.

**Figure 2:**
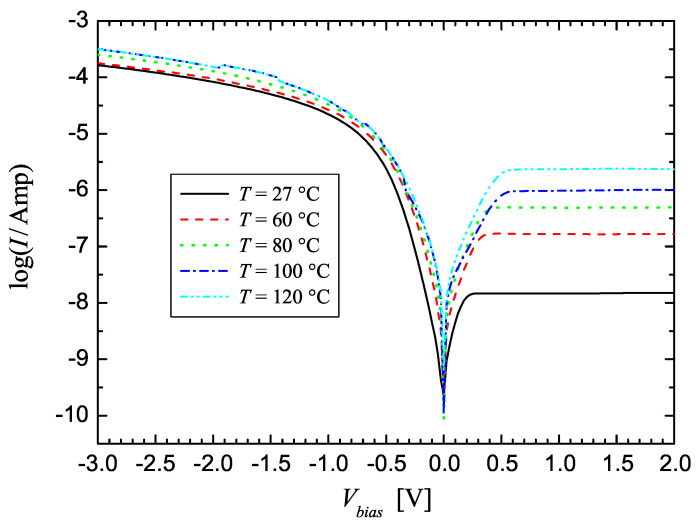
Semi-logarithmic plot of the current–voltage characteristic for a graphene/*p*-silicon diode measured at different temperatures.

Figure 2 shows the measured *I*-*V* curves for a Gr-Si Schottky diode obtained by varying the temperature from 27 °C to 120 °C. Increasing the temperature, we can observe an increase in the diode reverse current, because more electrons gain sufficient thermal energy to surmount the Schottky barrier, while the effect of temperature on the forward current is less evident, because for high forward voltages, the exponential dependence from the applied bias is absent, and the effect of the series resistance becomes more important.

## 4. Data Analysis

### 4.1. Extraction of Parameters via the Richardson Plot

A common protocol adopted for the extraction of the main diode parameters relies on the analysis of the *I* vs. $V_{bias}$ measurements in terms of Equations (1) and (2). The ideality index, together with the zero-bias SBH, are obtained by fitting the experimental data at different temperatures, for small direct polarizations, say ∼−0.1 V $\leq V_{bias}\leq 0$ V [2], with the expression

(19) $$\ln\left[I{\left(1-e^{\;qV_{bias}/k_{B}T}\right)}^{-1}\right]≃\ln I_{0}-\frac{qV_{bias}}{n\;k_{B}T}$$

Using this approach, the effects of the surface states are disregarded but implicitly encompassed in the extracted parameters.

**Figure 3:**
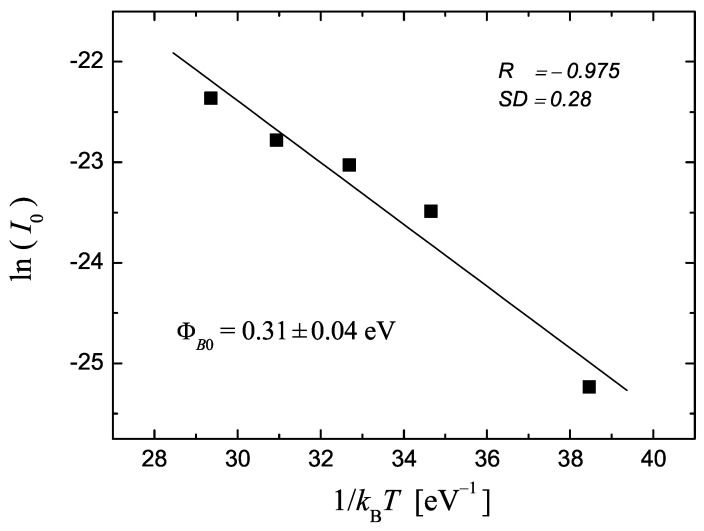
Linear fitting of $\ln I_{0}$ vs. $1/k_{B}T$ for the I5 device. *R* and SD relate to the quality of the fitting; $\Phi_{B0}$ is the resulting zero-bias SBH extracted from the fitting.

Figure 3 shows the $\ln I_{0}$ vs. $1/k_{B}T$ plot (Richardson plot) for the I5 device, from which $\Phi_{B0}$, $\langle n\rangle$ and $\langle A^{*}\rangle$ are extracted. The values obtained from different diodes are reported in Table 1.

The relation $I_{0}\propto T^{2}$ is only appropriate for metal/semiconductor diodes and not for Gr/Si ones [4,5,6]; we nevertheless decided to maintain it, since (i) the details of the pre-exponential factors are marginal at this stage, and (ii) the contrast of the worked out values with the metal/semiconductor case is better highlighted.

It is important to note that, e.g., in the case of the D3 device, $\Phi_{B0}\approx \delta E$. However, δE varies between 0.24 eV and 0.32 eV upon changing the temperature from 27 °C to 120 °C (cf. Equation (9)); this would point to an almost zero or even negative $\phi_{bi}$ (cf. Equation (13)), that is, a condition where the ohmic regime would be entered, in contrast with the spirit in which $\Phi_{B0}$ was extracted. The low values of $\Phi_{B0}$, as well as those found for the ideality index (≈2), can be explained considering the effect of silicon surface states.

### 4.2. I-V Fitting Including Surface Effects

#### 4.2.1. Thermionic Regime: $\Phi_{B0}$ and the Ideality Index *n*

The *I* vs. $V_{bias}$ experimental data are fitted for *each single T* with the equation

(20) $$I=A\;A^{*}T^{2}e^{-\Psi_{B}\left(V_{bias}\right)/nk_{B}T}\left[1-e^{qV_{bias}/k_{B}T}\right]\;,$$

from which Equations (1) and (2) can be obtained upon expanding $\Psi_{B}\left(V_{bias}\right)$ to first order in $V_{bias}$; in this respect, it is worth noticing that the zero-bias SBH of Equation (2), $\Phi_{B0}$, would now correspond to ${\left.\Psi_{B}\right|}_{V_{bias}=0}/n$. We would underline that the parameter *n* is now the *residual* ideality index after all the effects considered so far are encompassed in $\Psi_{B}$, such as the $\phi_{bi}\left(V_{bias}\right)$ dependence and the effect of surface states; the image charge correction is disregarded, and this would affect *n* by just about a 5% (cf., e.g., ref. [22]).

Assuming the presence of an oxide layer with thickness $d_{i}=1$ nm, and setting $n_{0}\approx 10^{13}$ ${\mathrm{cm}}^{-2}$, after Hall measurements, we estimate from Equation (8) (with the condition $\phi_{bi}=0$) that a transition from the thermionic to ohmic regime is expected to occur around the flat band potential $V_{FB}\approx -0.1$ V.

**Figure 4:**
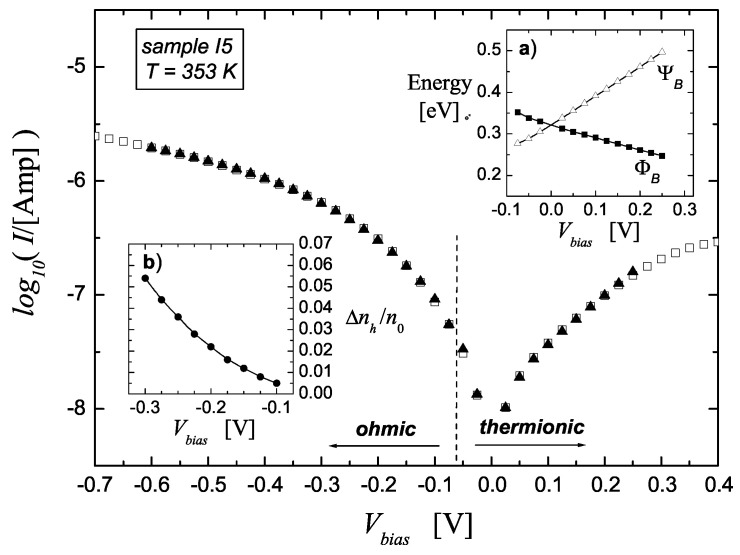
Fitting of the experimental $I-V_{bias}$ data (open squares) with Equation (20) in thermionic regime and with Equation (18) in ohmic regime (black triangles) for sample I5 at T=80 °C. The Gr carrier density is set to $n_{0}=10^{13}$ ${\mathrm{cm}}^{-2}$. The worked out parameters are reported in Table 2. Inset (**a**) shows the $V_{bias}$ dependencies of the backward SBH, $\Phi_{B}$ (filled squares), and forward SBH, $\Psi_{B}$ (open triangles), within the thermionic regime; inset (**b**) reports the ratio $\Delta n_{h}/n_{0}$ in the ohmic regime.

Table 2 collects the relevant parameters for diode I5 extracted from the fitting of *I*-*V* curves at different temperatures; an example of how the fittings look is shown in Figure 4 for the I5 device at T=80 °C. In all cases, as well as in those collected in Table 3, a good matching between thermionic and ohmic regimes was always found for a flat band potential $V_{FB}≃-0.07÷-0.11$ V, which is very close to what was expected (fluctuations around this value were found particularly in devices with small area). It is noteworthy that the typical $D_{i}$ and $\Delta E_{0}$ worked out values are rather similar to those found for Si-oxide/Si interfaces by capacitance measurements [23].

The $\Phi_{B}$ and $\Psi_{B}$ vs. $V_{bias}$ dependencies are almost linear, as shown by inset *a* in Figure 4; this legitimates a posteriori the first order Taylor approximation of $\Psi_{B}\left(V\right)$ in Equation (1). All fittings were done in the interval −0.6 V $\leq V_{bias}≲0.1÷0.3$ V. The zero-bias SBH values reported in Table 2 are linear interpolations from the $\Phi_{B}$ vs. $V_{bias}$ dependence and are derived at each *T*.

The chi-square values, $X^{2}$, reported in Table 2 and Table 3 refer to the thermionic regime fitting domain, which is most critical. In fact, one proceeds by first considering the *I*-*V* patterns in the thermionic domain to derive parameters such as $D_{i}$, $\Delta E_{0}$ and *n* via a non-linear regression. Once the latter are determined, *J* and α in the ohmic domain can be worked out without particular problems, given their reduced number. This procedure is repeated iteratively, meanwhile also adjusting $V_{FB}$ for better matching the fitting curves at the border between the ohmic and thermionic regions. Good quality fittings in the thermionic domain are compulsory; for this reason, the associated $X^{2}$ values are highlighted.

As expected, by introducing the effect of silicon surface states in the fitting model, the worked out $\Phi_{B0}$’s are found to be a bit larger than those obtained from the Richardson analysis (cf. Table 1). Moreover, the *n* values now extracted are very close to unity, indicating a quite ideal behavior, and the inequality $\Phi_{B0}\left(T\right)>\delta E\left(T\right)$ always holds. This evidence supports the idea of the important role played by silicon surface states in the current conduction of Gr/*p*-Si junctions.

Considering the Richardson constant, the fitting values obtained are around $A^{*}\approx 5\times 10^{-6}$ Amp ${\mathrm{cm}}^{-2}$ $K^{-2}$, very similar to those reported in Table 1. Thus, for a temperature T≈350 K, one has $A^{*}T^{2}\approx 0.6$ Amp ${\mathrm{cm}}^{-2}$. Assuming $l_{⊥}\approx 0.5$ nm, we find $\lambda T\;v_{⊥}\approx 0.02$ cm/s (cf. Equation (7)).

On the other hand, the initial out-of-plane velocity of a scattered Gr electron which has a chance to tunnel towards *p*-Si is in the order of $v_{⊥}≲\Delta E/p_{⊥}$ [10], that is (since $p_{⊥}∼\Delta p_{⊥}∼\hbar /\Delta r_{⊥}$), $v_{⊥}≲10^{5}$ cm/s. Then, by taking λ≈0.1, i.e., assuming about the same chances for parallel momentum-conserving and non-conserving collisions, we would obtain a transmission coefficient $T\approx 10^{-6}$. As a consequence, by letting $d_{i}=1$ nm, one finds ΔU≡U−ϵ≃0.47 eV; this energy barrier would lower on accounting properly for the fraction of collisions which do not conserve the in-plane momentum.

#### 4.2.2. Ohmic Regime

From Table 2 and Table 3, one sees that α≈1. In the first approximation, this points at a linear dependence of *I* on the electric field (cf. Equation (17)):

(21) $$I\approx A\;J\;\frac{\Delta n_{h}}{n_{0}}\propto {\left.E\right|}_{x=0}\;.$$

To provide a connection with the actual form of the saturation current, we resort to continuity:

(22) $$J\;{\left(\frac{\Delta n_{h}}{n_{0}}\right)}^{\alpha }=q\;n_{e}v_{⊥}\;,$$

where $n_{e}$ is the density of Gr electrons that are *allowed to tunnel* through the oxide layer located between Gr and Si, and $v_{⊥}$ is their normal velocity on impinging the Gr/oxide interface. From the previous arguments of the Theory section,

(23) $$n_{e}\approx \frac{n_{0}}{l_{⊥}}\lambda \;T$$

and, considering a typical value of $J∼5\times 10^{-3}$ Amp ${\mathrm{cm}}^{-2}$ as from Table 2 and Table 3, one finds $\lambda \;T\;v_{⊥}∼10^{-4}$ cm/s, i.e., about two orders of magnitude less than in the thermionic case.

To explain this low value, we suggest that surface states may possibly play a role. In fact, as anticipated in the theory section, within the thermionic regime, they are mostly filled in, while in the transition to the ohmic regime, they progressively rise above $E_{Fs}$ and tend to ionize. The tunneled electrons would get temporarily anchored to these empty states, until the electric field will drag them away. This circumstance would be responsible for some artifact in the Richardson procedure for the estimate of the diode parameters, at least when $\left|V_{FB}\right|$ values are small.

The capture of these electrons does not seem to be so rare an event. Consider as an example the I5 device in direct polarization at $V_{bias}=-0.3$ V (cf. Figure 4 and Table 2); then, $\Delta n_{h}≃5\times 10^{-2}\;n_{0}$ and, from Equation (16), $\phi_{bi}≃-0.2$ V and ${\left|E\right|}_{x=0}\approx 10^{4}$ V/cm, while the surface density of the localized surface states is in the order of $D_{i}\Delta E_{0}\approx 5\times 10^{11}$ ${\mathrm{cm}}^{-2}$. On the other hand, assuming $v_{⊥}∼10^{5}$ cm/s, one finds $n_{e}l_{⊥}∼10^{4}$ ${\mathrm{cm}}^{-2}$ in the ohmic regime, which is much smaller than $D_{i}\Delta E_{0}$.

In these circumstances, a corrected expression of the overall transmission coefficient should be adopted for the appropriate description of the ohmic regime, including both the effects of traps and electric field.

## 5. Concluding Remarks

The extraction of the Gr/*p*-Si junction parameters from the current–voltage–temperature characteristics in the thermionic regime usually gives a large ideality index and a small value for the potential barrier of the diodes. Generally, an ideality index larger than unity is related to the deviation from pure thermionic emissions as well as to inhomogeneities of the barrier height. But we show that Gr/*p*-Si diodes work as quite ideal junctions when taking into account the bias dependence of the SBH in the fitting model, as well as the effect played by silicon surface states. In fact, our approach showed that the values obtained for these parameters are significantly influenced by the silicon surface states on the current conduction. The role of these states in the electrical properties of MOS devices is well known, and we can say that they are present in Gr/Si diodes, probably also for the weak Van der Waals interaction between Gr and Si.

The comparison of the electron tunneling efficiencies in the thermionic and ohmic regimes turned out to be crucial for highlighting the impact of the localized states in the characteristic features of these 2D/3D Schottky devices, and to our knowledge, this strategy has not been followed before in the literature.

The approximate expression derived for a saturation current appropriate for our devices pointed out the product $\lambda \;T\;v_{⊥}$ as a key indicator of their electrical performances. Irrespective of the detailed form of the transmission coefficient T, we referred to this quantity in the discussion of the data. In turn, the results suggested that the modeling of T as proposed in the theory section is incomplete, in the sense that the presence of trap states at the Si surface, as well as of an electric field (rather strong as we saw) which both modulates the profile of the oxide potential barrier and de-traps electrons from these localized states, must be envisaged. These aspects cannot be overlooked, since $\lambda \;T\;v_{⊥}$ changes of about two orders of magnitude are revealed by the analysis; for this reason, a better suited form of T is actually being considered. This may also be worth when estimating the $v_{⊥}$ drop in crossing the interface. Certainly such effects are “felt” by the phenomenological model of Equation (18), although not explicitly highlighted, and further work has to be done in this respect.

Finally, we explicitly found through our quantum estimates that the $T^{1}$ scaling of the saturation current also holds in our case, confirming that this feature depends on the vertical architecture of these devices and not on the nature of the charge carriers inherent to the semiconductor moiety.

In conclusion, the model presented in the paper provides insight into the physical mechanism governing the Gr/Si junction operation, such as charge transport, carrier injection and interface effects, which are fundamental in further improving the device performance and exploring new device functionalities. This is fundamental at this stage, when the integration of graphene and related layered materials in the semiconductor platform is becoming true via the Experimental 2D Pilot Line, which offers prototyping services to companies, research centers and academics to develop their innovative technologies based on 2D materials in an established processing platform.

## Figures and Tables

**Table 1:** Parameters extracted from the Richardson plots for different devices. *A* (in $10^{-3}$ ${\mathrm{cm}}^{2}$) is the area of the diode, $\langle n\rangle$ is the *T*-average ideality index, $\langle A^{*}\rangle$ (in $10^{-6}$ Amp ${\mathrm{cm}}^{-2}$ $K^{-2}$) is the *T*-average Richardson constant, and $\Phi_{B0}$ (in eV) is the zero-bias SBH as obtained from the linear regressions on the *I* vs. $V_{bias}$ data (cf. Equation (19)) at temperatures T=27,60,80,100 and 120 °C.

Device	*A*	$\langle n\rangle$	$\langle A^{*}\rangle$	$\Phi_{B0}$
A3	7.85	2.1	3.0	0.26±0.03
D3	6.36	1.9	2.0	0.25±0.04
E4	3.85	1.8	2.2	0.28±0.04
G5	1.96	1.7	6.0	0.31±0.03
I5	1.26	1.8	1.7	0.31±0.04

**Table 2:** Fitting parameters for the I5 device at different temperatures *T* (in °C) as obtained from the analysis with the complete model illustrated in the Theory Section. In the columns, $D_{i}$ (in $10^{12}$ ${\mathrm{eV}}^{-1}$ ${\mathrm{cm}}^{-2}$) is the surface states’ density, $\Delta E_{0}\equiv {\left[q\phi_{0}-E_{Fs}\right]}_{FB}$ (in eV) is the upper limit of the surface states excess energy with respect to $E_{Fs}$ in the absence of band curvature, *n* is the ideality index, $A^{*}$ (in $10^{-6}$ Amp ${\mathrm{cm}}^{-2}$ $K^{-2}$) is the Richardson constant, $\Phi_{B0}$ (in eV) is the zero-bias SBH, $X^{2}$ (in units of $10^{-3}$) is the chi-square value limited to the fitting within the thermionic regime interval, *J* is a fitting parameter in $10^{-3}$ Amp ${\mathrm{cm}}^{-2}$, and α is a fitting exponent (cf. Equation (18)).

*T*	$D_{i}$	$\Delta E_{0}$	*n*	$A^{*}$	$\Phi_{B0}$	$X^{2}$	*J*	α
27	2.5 ± 0.2	0.18	1.1 ± 0.03	1.2	0.30	1.5	5.4	1.08
60	2.6	0.22	1.1	4.2	0.32	3.3	86.1	1.40
80	2.5 ± 0.1	0.12	1.02	3.2 ± 0.4	0.32	1.9	5.9 ± 0.4	0.84
100	3.0 ± 0.8	0.12 ± 0.06	1.06	2.5	0.32	3.2	5.9	0.84
120	1.8	0.30	1.10	3.2	0.34	0.5	28	1.07

**Table 3:** Same as Table 2 for the A3, D3, E4, G5, I5 devices at a temperature T=80 °C.

Device	$D_{i}$	$\Delta E_{0}$	*n*	$A^{*}$	$\Phi_{B0}$	$X^{2}$	*J*	α
A3	2.3 ± 0.3	0.11	1.01	3.4 ± 0.1	0.32	4.3	2.9 ± 0.1	0.73
D3	2.4 ± 0.3	0.11 ± 0.02	1.02 ± 0.25	5.3 ± 0.3	0.32	3.0	4.8 ± 0.1	0.73
E4	1.80	0.18	1.08	2.5	0.31	2.0	2.3 ± 0.3	1.07
G5	2.5 ± 0.1	0.13	1.02	9.7 ± 0.5	0.33	1.4	9.6 ± 0.4	1.18
I5	2.5 ± 0.1	0.12	1.02	3.2 ± 0.4	0.32	1.9	5.9 ± 0.4	0.84

## Data Availability

Data are contained within the article.
